# The influenza pandemic preparedness planning tool *InfluSim*

**DOI:** 10.1186/1471-2334-7-17

**Published:** 2007-03-13

**Authors:** Martin Eichner, Markus Schwehm, Hans-Peter Duerr, Stefan O Brockmann

**Affiliations:** 1 Department of Medical Biometry, University of Tübingen, Germany; 2 Baden-Württemberg State Health Office, District Government Stuttgart, Germany

## Abstract

**Background:**

Planning public health responses against pandemic influenza relies on predictive models by which the impact of different intervention strategies can be evaluated. Research has to date rather focused on producing predictions for certain localities or under specific conditions, than on designing a publicly available planning tool which can be applied by public health administrations. Here, we provide such a tool which is reproducible by an explicitly formulated structure and designed to operate with an optimal combination of the competing requirements of precision, realism and generality.

**Results:**

*InfluSim *is a deterministic compartment model based on a system of over 1,000 differential equations which extend the classic SEIR model by clinical and demographic parameters relevant for pandemic preparedness planning. It allows for producing time courses and cumulative numbers of influenza cases, outpatient visits, applied antiviral treatment doses, hospitalizations, deaths and work days lost due to sickness, all of which may be associated with economic aspects. The software is programmed in Java, operates platform independent and can be executed on regular desktop computers.

**Conclusion:**

*InfluSim *is an online available software http://www.influsim.info which efficiently assists public health planners in designing optimal interventions against pandemic influenza. It can reproduce the infection dynamics of pandemic influenza like complex computer simulations while offering at the same time reproducibility, higher computational performance and better operability.

## Background

Preparedness against pandemic influenza has become a high priority public health issue and many countries that have pandemic preparedness plans [1]. For the design of such plans, mathematical models and computer simulations play an essential role because they allow to predict and compare the effects of different intervention strategies [2]. The outstanding significance of the tools for purposes of intervention optimization is limited by the fact that they cannot maximize realism, generality and precision at the same time [3]. Public health planners, on the other hand, wish to have an optimal combination of these properties, because they need to formulate intervention strategies which can be generalized into recommendations, but are sufficiently realistic and precise to satisfy public health requirements.

Published influenza models which came into application, are represented by two extremes: generalized but over-simplified models without dynamic structure which are publicly available (e.g. [4]), and complex computer simulations which are specifically adjusted to real conditions and/or are not publicly available (e.g. [5,6]). The complexity of the latter simulations, however, is not necessary for a reliable description of infection dynamics in large populations [7]. A minimum requirement for a pandemic influenza planning tool is a dynamic modelling structure which allows investigation of time-dependent variables like incidence, height of the epidemic peak, antiviral availability etc. The tool should, on the other hand, be adjustable to local conditions to adequately support the pandemic preparedness plans of different countries which involve considerably different assumptions (Table 1).

**Table 1:** Pandemic preparedness plans of some countries

	Attack rate	Outpatients per 100.000 population	Hospitalizations per 100.000 population	Deaths per 100.000 population	Reference
Germany	15%	15,859	437	117	[9]
USA					
- moderate	30%*	15,000	320	77	[31]
- severe	30%*	15,000	3,666	705	[31]
- CDC	35%*	17,718	277	78	[4]
GB	25%	25,000	140	90	[32]
France	25%	25,000	99	20	[33]
Netherlands	30%	30,000	64	26	[34], [35]
Japan	25%*	13,077	41	13	[36]
Canada	35%*	16,066	359	137	[37]

Assumed scenarios and outcomes of pandemic preparedness plans. * Gross attack rate (i.e. clinically ill and moderately ill cases).

Here we describe a publicly available influenza pandemic preparedness planning tool [8] which is designed to meet the requirements in preparedness planning. It is based on an explicitly formulated dynamic system which allows addressing time-dependent factors. It is sufficiently flexible to evaluate the impact of most candidate interventions and to consider local conditions like demographic and economic factors, contact patterns or constraints within the public health system. In subsequent papers we will also provide examples and applications of this model for various interventions, like antiviral treatment and social distancing measures.

## Implementation

The model is based on a system of 1,081 differential equations which extend the classic SEIR model. Demographic parameters reflect the situation in Germany in 2005, but can be adjusted to other countries. Epidemiologic and clinic values were taken from the literature (see Tables 1, 2, 3, 4, 5, 6 and the sources quoted there). Pre-set values can be varied by sliders and input fields to make different assumptions on the transmissibility and clinical severity of a new pandemic strain, to change the costs connected to medical treatment or work loss, or to simply apply the simulation to different demographic settings. Model properties can be summarized as follows. The mathematical formulation of this model is presented in detail in the online supporting material. The corresponding source code, programmed in Java, and further information can be downloaded from [8].

**Table 2:** Age distribution and risk categories

	children			working adults		elderly
	
	0–5	6–12	13–19	20–39	40–59	60 +
Population size *N*_*a*_	5,272	6,773	7,952	25,959	29,127	24,917

A population of *N *= 100,000 inhabitants of Germany is subdivided according to age *a *and risk category *r*. We assume that all age groups are fully susceptible at begin of the outbreak. A fraction of *F*_*a *_= 6% of all children (age < 20 years) are regarded as being under high risk (*r *= *r*_1_) after an influenza infection whereby the remaining 94% are under low risk (*r *= *r*_2_). The high risk fractions of working adults (ages 20–59) and elderly (ages 60+) are *F*_*a *_= 14% and *F*_*a *_= 47%, respectively. Source: [9]

**Table 3:** WAIFW matrix

	0–5	6–12	13–19	20–39	40–59	60 +
0–5	169.14	31.47	17.76	34.50	15.83	11.47
6–12	31.47	274.51	32.31	34.86	20.61	11.50
13–19	17.76	32.31	224.25	50.75	37.52	14.96
20–39	34.50	34.86	50.75	75.66	49.45	25.08
40–59	15.83	20.61	37.52	49.45	61.26	32.99
60 +	11.47	11.50	14.96	25.08	32.99	54.23

The who-acquires-infection-from-whom matrix $K_{a_{s},a_{i}}$ shows the frequency of contacts (per week per person) between different age classes. Source: [38].

**Table 4:** Sojourn times

Period	average duration	stages	coefficient of variation
Latent period	*D*_*E *_= 1.9 days ^*A*^	*n *= 7	37.8% ^*A*^
Fully contagious period			
asymptomatic and moderately sick adults	4.1 days ^*A*^	*m *= 19	22.9% ^*A*^
others	7.0 days ^*B*^	*m *= 19	22.9% ^*C*^
Period of convalescence	*D*_*R *_= 5 days ^*D*^	*j *= 9	33.3% ^*C*^

Distribution of sojourn times (the last two stages of the latent period are used as early infectious period with an average duration of *D*_*L *_= 0.5 days). Sources:^*A *^[11], ^*B *^[39, 40], ^*C *^assumed, ^*D *^[41]

**Table 5:** Clinical course

	under 20	20 to 59	60 and older
Hospitalized fraction *h*_*a*, *r *_of untreated severe cases			
low risk group (*r *= *r*_1_)	0.187%	2.339%	3.560%
high risk group (*r *= *r*_2_)	1.333%	2.762%	7.768%

Case fatality *d*_*a *_of hospitalized cases	5.541%	16.531%	39.505%

Independent of age *a *and risk group *r*, a fraction *c*_*a*, *r *_(*A*) = 33% of infections result in asymptomatic cases, a fraction *c*_*a*, *r *_(*M*) = 33.5% become moderately sick and the remaining fraction develops severe disease. An age- and risk-dependent fraction *h*_*a*, *r *_of untreated patients with severe disease needs hospitalization. An age-dependent fraction *d*_*a *_of hospitalized cases dies. Sources: fraction of asymptomatic cases: [11]; 50% of symptomatic cases see a doctor: [9]; hospitalizations per severe case: [9]; case fatality of hospitalized, but untreated patients calculated from [4].

**Table 6:** Contagiousness

Basic reproduction number	*R*_0 _= 2.5
Relative contagiousness during the early infectious phase	*b*_*L *_= 50%
Relative contagiousness of asymptomatic cases	*b*_*A *_= 50%
Relative contagiousness of moderately sick cases	*b*_*M *_= 100%
Relative contagiousness of very sick cases	*b*_*V *_= 100%
Concentration of the cumulative contagiousness during the first half of the symptomatic period	*x*_50 _= 90%

Sources: Contagiousness of asymptomatic cases: [11]; degree of contagiousness during the early infectious period and equality of the contagiousness of moderately and severely sick cases: assumed.

According to the German National Pandemic Preparedness Plan [9], the total population is divided in age classes, each of which is subdivided into individuals of low and high risk (Table 2). Transmission between these age classes is based on a contact matrix (Table 3) which is scaled such that the model with standard parameter values yields a given basic reproduction number *R*_0_. Values for the *R*_0 _associated with an influenza strain with pandemic potential are suggested to lie between 2 and 3 [10]. This value is higher than the effective reproduction number which has been estimated to be slightly lower than 2 [11,12]. As a standard parameter, we use *R*_0 _= 2.5 which means that cases infect on average 2.5 individuals if everybody is susceptible and if no interventions are performed.

Susceptible individuals who become infected, incubate the infection, then become fully contagious and finally develop protective immunity (Table 4). A fraction of cases remains asymptomatic; others become moderately sick or clinically ill (i.e. they need medical help). Depending on the combination of age and risk group, a fraction of the clinically ill cases needs to be hospitalized, and an age-dependent fraction of hospitalized cases may die from the disease (Table 5). This partitioning of the cases into four categories allows combining the realistic description of the transmission dynamics with an easy calculation of the resources consumed during an outbreak. The degree and duration of contagiousness of a patient depend on the course of the disease; the latter furthermore depends on the age of the patient (Table 5). Passing through the incubation and contagious period is modelled in several stages which allows for realistic distributions of the sojourn times (Table 4). The last two stages of the incubation period are used as early infectious period during which the patient can already spread the disease. Infectiousness is highest after onset of symptoms and thereafter declines geometrically (Table 6). Clinically ill patients seek medical help on average one day after onset of symptoms. Very sick patients are advised to withdraw to their home until their disease is over, whereas extremely sick patients need to be hospitalized and may die from the disease (Table 4). After the end of their contagious period, clinically ill patients go through a convalescent period before they can resume their ordinary life and go back to work (Table 4).

## Results

**Figure 1:**
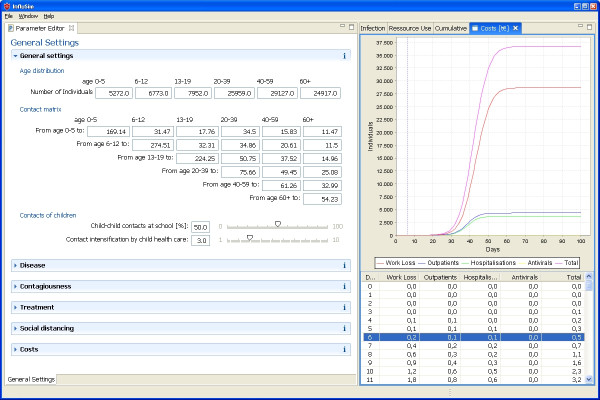
***InfluSim *user interface**. Graphical user interface of *InfluSim*. Parameter values can be varied within different tabs (left hand side), divided into *General settings *(demography by age and risk group, contact matrix, economics), *Disease *(sojourn times, symptoms, hospitalizations, case fatality), *Contagiousness *(*R*_0_, infectivity over time and by disease severity), *Treatment *(therapeutic window, treatment schedules, antiviral properties), *Social distancing *(isolation schedules, general contact reduction, closing day care centres and schools, cancelling mass gatherings) and *Costs *(work loss, hospitalization, treatment). Time-dependent model output (right hand side) visualizes *Infection *prevalence (susceptible, exposed, asymptomatic, moderately sick, severely sick, dead, immune), *Resource use *(work loss, outpatients, hospital beds, antivirals), *Cumulative *numbers of the latter, and *Costs*.

**Figure 2:**
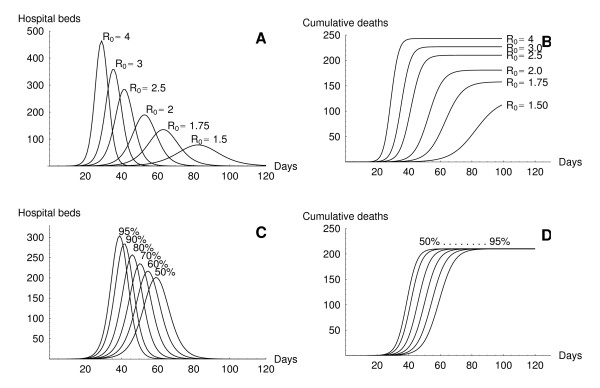
***InfluSim *output**. Examples of *InfluSim *output for a population of 100,000 citizens. **A**: Number of hospital beds required during an influenza pandemic for values of *R*_0 _∈ {1.5, 1.75, 2, 2.5, 3, 4}. **B**: Cumulative number of deaths for values of *R*_0 _as in A. **C**: Number of hospital beds for values of *x*_50 _∈ {50, 60, 70, 80, 90, 95%} (e.g. *x*_50 _= 95% means that 95% of the cumulative contagiousness is concentrated during the first half of the contagious period, see Table 6). **D**: Cumulative number of deaths for values of *x*_50 _as in C. All other parameters as listed in Tables 2-6.

We provide some examples of model output of *InfluSim *[8], version 2.0, by means of four sensitivity analyses; further investigations will be presented elsewhere. Figure 1 shows the graphical user interface of the software which is divided into input and output windows. The user may set new values in the input fields or move sliders to almost simultaneously obtain new results for the course of an epidemic in a given population. Figures 2A and 2B show pandemic waves which result from varying the basic reproduction number from 1.5 to 4.0. Using the standard parameter values as given in Tables 2, 3, 4, 5, 6 and omitting all interventions in a town of 100,000 inhabitants results in a pandemic wave which lasts for about ten weeks (Figure 2A, with *R*_0 _= 2.5). The peak of the pandemic wave is reached after six to seven weeks, with a daily incidence of up to 2,340 influenza patients seeking medical help, with up to 280 hospital beds occupied by influenza cases and with up to 14,000 out of 60,000 working adults unable to go to work because of illness or convalescence. These results depend on the assumptions concerning the yet unknown contagiousness and pathogenicity of the virus. Figures 2C and 2D show how the shape of the curves depends on the course of contagiousness: the pandemic wave proceeds relative slowly if the contagiousness does not change during the infectious period (*x*_50 _= 50%), but proceeds quickly if the contagiousness is highest after onset of symptoms and decreases thereafter (*x*_50 _> 50%).

## Discussion and Conclusion

The influenza pandemic preparedness planning tool *InfluSim *stands between simple spreadsheet models and sophisticated stochastic computer simulations. It describes a pandemic wave within a homogeneously mixing population like a town or city, but surprisingly produces the same dynamics as individual-based simulations which explicitly consider geographic spread through the US (cf. [6] and [5] with Figure 2 using *R*_0 _= 2). Similar observations were made with a simple deterministic compartmental model [7]. Stochastic models are known to behave quasi-deterministically when the simulated population becomes very large.

A further reason for the congruence of complex stochastic and simple deterministic models must lie in the incredibly quick way in which pandemic influenza spreads geographically. Unless being controlled at the place of origin [12,13], a pandemic starting in a far-off country will lead to multiple introductions [14] into the large industrialized nations where it can be expected to quickly spread to neighbouring towns and to rural areas. The large populations which have to be considered susceptible to a pandemic virus and the quick geographic spread tend to diminish the differences between the results of sophisticated individual-based and simple deterministic models.

However, a deterministic model like *InfluSim *cannot reliably represent effects originating from stochasticity, from effects in small populations, or from heterogeneities. Examples are: (i) a geographically limited spread and fairly effective control measures can imply that the epidemic affects only a small population and thus, may be strongly influenced by stochastic events [15-17]; (ii) transmission which predominantly occurs in households or hospitals, or which is driven by other substantial features of the contact network is not in agreement with the assumption of homogeneous mixing in the deterministic model cannot reliably predict the spread of infection [18-23]. In particular, (iii) super-spreading events can substantially change the course of an epidemic compared to the deterministic prediction [24-27]. Apart from such factors, the predictability of intervention success is generally subject to uncertainties in the choice of parameter values, demanding additional efforts like Bayesian approaches [28] to evaluate the reliability of predictions [29].

Pandemic preparedness plans must consider constraints and capacities of locally operating public health systems. The time-dependent solutions of *InfluSim *allow assessing peak values of the relevant variables, such as outpatients, hospitalizations and deaths. Various interventions may be combined to find optimal ways to reduce the total number of cases, to lower the peak values or to delay the peak, hoping that at least part of the population may benefit from a newly developed vaccine.

Special care was taken when implementing a variety of pharmaceutical and non-pharmaceutical interventions which will be discussed in subsequent papers. Despite its comprehensible structure, the model does not suffer from over-simplifications common to usual compartment models. Instead of implicitly using exponentially distributed sojourn times, we have implemented realistically distributed delays. For example, the model considers that individuals may transmit infection before onset of symptoms, and that some cases may remain asymptomatic, but still infecting others. Such features have serious implications for the success of targeted control measures.

*InfluSim *is freely accessible, runs on a regular desktop computer and produces results within a second after changing parameter values. The user-friendly interface and the ease at which results can be generated make this program a useful public health planning tool. Although we have taken care of providing a bug-free program, including the source code, the user is encouraged to treat results with due caution, to test it, and to participate in bug-reports and discussions on the open-source platform [30] which also provides regular updates of *InfluSim*.

## Availability and requirements

Project name: InfluSim version 2.0

Project home page: http://www.influsim.info

Sourceforge: http://sourceforge.net/projects/influsim

Operating systems: Platform independent

Programming language: Java

Other requirements: e.g. Java 1.5 or higher

License: CPL

Any restrictions to use by non-academics: none

## Competing interests

The author(s) declare that they have no competing interests.

## Authors' contributions

ME developed the model, MS designed the software, HPD wrote the manuscript and SOB formulated the public health requirements of the software. All authors read and approved the final manuscript.

## Appendix: Description of the transmission dynamics of InfluSim version 2.0

Susceptible individuals *S*_*a*, *r *_are infected at a rate *λ*_*a*_(*t*) which depends on their age *a *and on time *t*. Infected individuals, *E*_*a*, *r*_, incubate the infection for a mean duration *D*_*E*_. To obtain a realistic distribution of this duration, the incubation period is modelled in *n *stages so that progression from one stage to the next one occurs at rate *δ *= *n*/*D*_*E*_. The last *l *incubation stages are regarded as early infectious period during which patients may already spread the infection (this accounts for an average time of *lD*_*E*_/*n *for the "early infectious period" which is about half a day for the standard set of parameters). After passing through the last incubation stage, infected individuals become fully contagious and a fraction of them develops clinical symptoms. The course of disease depends on the age *a *of the infected individual and on the risk category *r *to which he or she belongs: a fraction *c*_*a*, *r*_(*A*) becomes asymptomatic (*A*_*a*_), a fraction *c*_*a*, *r *_(*M*) becomes moderately sick (*M*_*a*_), a fraction *c*_*a*, *r *_(*V*) becomes very sick (*V*_*a*_) and the remaining fraction *c*_*a*, *r *_(*X*) becomes extremely sick (*X*_*a*_) and need hospitalization (i.e., *c*_*a*, *r*_(*A*) + *c*_*a*, *r *_(*M*) + *c*_*a*, *r *_(*V*) + *c*_*a*, *r *_(*X*) = 1 for each combination of *a *and *r*). The rationale for distinguishing very sick and extremely sick cases is that only extremely sick cases can die from the disease and need to be hospitalized; in all other aspects, both groups of severe cases are assumed to be identical. The duration of the fully contagious stage depends on the course of the disease and on the age of the case. Sojourn times are *D*_*A*, *a *_and *D*_*M*, *a *_for asymptomatic and moderately sick cases, respectively, and *D*_*V*, *a *_for both groups of severe cases. To obtain realistic distributions of these sojourn times, the contagious classes are modelled in *m *stages each so that progression from one stage to the next occurs at rate *γ*_*A*, *a *_= *m*/*D*_*A*, *a*_, *γ*_*M*, *a *_= *m*/*D*_*M*, *a *_and *γ*_*V*, *a*, *U *_= *m*/*D*_*V*, *a*_, respectively. Severe cases seek medical help on average *D*_*D *_days after onset. Assuming that the waiting time until visiting a doctor is exponentially distributed, we use a constant rate *α *= 1/*D*_*D *_for doctoral visits. Very sick patients (*V*_*a*_) who visit a doctor are advised to withdraw to their home (*W*_*a*_) until the disease is over whereas extremely sick cases (*X*_*a*_) are immediately hospitalized (*H*_*a*_). A fraction *f*_*V *_(*t*) of all severe and a fraction *f*_*X *_(*t*) of all extremely severe cases who visit the doctor within *D*_*T *_days after onset of symptoms are offered antiviral treatment, given that its supply has not yet been exhausted. As our model does not explicitly consider the age of the disease (which would demand partial differential equations), we use the contagious stages to measure time since onset and allow for treatment up to stage *m*_*a*, *T *_(see below for details). This imposes some variability to the maximum time until which treatment can be given, which may even improve the realism of the model with respect to real-life scenarios. Antiviral treatment reduces the patients' contagiousness by *f*_*I *_percent and it reduces hospitalization and death by *f*_*H *_percent. Extremely sick patients, whose hospitalization is prevented by treatment, are sent home and join the group of treated very sick patients(*W*_*a*, *T*_). The remaining duration of disease and contagiousness of treated cases is reduced by *f*_*D *_percent so that their rate of progressing from one stage to the next has to be changed to *γ*_*V*, *a*, *T *_= *m*/((1 - *f*_*D*_)*D*_*V*, *a*_). Extremely sick and hospitalized cases die at rates *τ*_*a*_, depending on their age *a*. Whereas asymptomatic (*A*_*a*_) and moderately sick patients (*M*_*a*_) who have passed their last stage of contagiousness are considered healthy immunes (*I*), very sick and extremely sick patients (classes *V*_*a*_, *W*_*a*, *U*_, *W*_*a*, *T*_, *X*_*a*_, *H*_*a*, *U *_and *H*_*a*, *T*_) first become convalescent (*C*_*a*_) for an average duration of *D*_*C *_days before they resume their ordinary life. To obtain a realistic distribution of this sojourn time, convalescence is modelled in *j *stages so that progression from one stage to the next occurs at rate *ρ *= *j*/*D*_*C*_. Fully recovered patients who have passed through their last stage of convalescence join the group of healthy immunes *I*; working adults will go back to work. Further interventions, describing the reduction of contacts, will be discussed after the presentation of the differential equations.

### Differential equation model describing the transmission dynamics

Susceptible individuals

${\dot{S}}_{a,r}=-\lambda_{a}(t)S_{a,r}$

Infected individuals who incubate the infection

$\begin{array}{cc} \begin{array}{l} {\dot{E}}_{1,a,r}=\lambda_{a}(t)S_{a,r}-\delta E_{1,a,r}\\ {\dot{E}}_{k,a,r}=\delta \left(E_{k-1,a,r}-E_{k,a,r}\right) \end{array} & \text{for }k=2,...,n \end{array}$

Asymptomatic infectious individuals

$\begin{array}{cc} \begin{array}{l} {\dot{A}}_{1,a}=\delta c_{a,r}(A)E_{n,a,r}-\gamma_{A,a}A_{1,a}\\ {\dot{A}}_{k,a}=\gamma_{A,a}\left(A_{k-1,a}-A_{k,a}\right) \end{array} & \text{for }k=2,...,m \end{array}$

Moderately sick individuals

$\begin{array}{cc} \begin{array}{l} {\dot{M}}_{1,a}=\delta c_{a,r}(M)E_{n,a,r}-\gamma_{M,a}M_{1,a}\\ {\dot{M}}_{k,a}=\gamma_{M,a}\left(M_{k-1,a}-M_{k,a}\right) \end{array} & \text{for }k=2,...,m \end{array}$

Very sick individuals who have not yet visited a doctor

$\begin{array}{cc} \begin{array}{l} {\dot{V}}_{1,a}=\delta c_{a,r}(V)E_{n,a,r}-\left(\gamma_{V,a,U}+\alpha \right)V_{1,a}\\ {\dot{V}}_{k,a}=\gamma_{V,a,U}\left(V_{k-1,a}-V_{k,a}\right)-\alpha V_{k,a} \end{array} & \text{for }k=2,...,m \end{array}$

Treated very sick individuals

$\begin{array}{ll} {\dot{W}}_{1,a,T}=\alpha \left(f_{V}(t)V_{1,a}+f_{X}(t)f_{H}X_{1,a}\right)-\gamma_{V,a,T}W_{1,a,T} & \\ {\dot{W}}_{k,a,T}=\alpha \left(f_{V}(t)V_{k,a}+f_{X}(t)f_{H}X_{k,a}\right)+\gamma_{V,a,T}\left(W_{k-1,a,T}-W_{k,a,T}\right) & \text{for }k=2,...,m_{a,T}\\ {\dot{W}}_{k,a,T}=\gamma_{V,a,T}\left(W_{k-1,a,T}-W_{k,a,T}\right) & \text{for }k=m_{a,T}+1,...,m \end{array}$

Untreated very sick individuals

$\begin{array}{ll} {\dot{W}}_{1,a,U}=\alpha \left(1-f_{V}(t)\right)V_{1,a}-\gamma_{V,a,U}W_{1,a,U} & \\ {\dot{W}}_{k,a,U}=\alpha \left(1-f_{V}(t)\right)V_{k,a}+\gamma_{V,a,U}\left(W_{k-1,a,U}-W_{k,a,U}\right) & \text{for }k=2,...,m_{a,T}\\ {\dot{W}}_{k,a,U}=\alpha V_{k,a}+\gamma_{V,a,U}\left(W_{k-1,a,U}-W_{k,a,U}\right) & \text{for }k=m_{a,T}+1,...,m \end{array}$

Extremely sick individuals who have not yet visited a doctor

$\begin{array}{cc} \begin{array}{l} {\dot{X}}_{1,a}=\delta c_{a,r}\left(X\right)E_{n,a,r}-\left(\gamma_{V,a}+\alpha +\tau_{a}\right)X_{1,a}\\ {\dot{X}}_{k,a}=\gamma_{V,a}\left(X_{k-1,a}-X_{k,a}\right)-\left(\alpha +\tau_{a}\right)X_{k,a} \end{array} & \text{for }k=2,...,m \end{array}$

Hospitalized and treated cases

$\begin{array}{ll} {\dot{H}}_{1,a,T}=\alpha f_{X}(t)\left(1-f_{H}\right)X_{1,a}-\left(\gamma_{V,a,T}+\tau_{a}\right)H_{1,a,T} & \\ {\dot{H}}_{k,a,T}=\alpha f_{X}(t)\left(1-f_{H}\right)X_{k,a}+\gamma_{V,a,T}\left(H_{k-1,a,T}-H_{k,a,T}\right)-\tau_{a}H_{k,a,T} & \text{for }k=2,...m_{a,T}\\ {\dot{H}}_{k,a,T}=\gamma_{V,a,T}\left(H_{k-1,a,T}-H_{k,a,T}\right)-\tau_{a}H_{k,a,T} & \text{for }k=m_{a,T}+1,...,m \end{array}$

Hospitalized, but untreated cases

$\begin{array}{ll} {\dot{H}}_{1,a,U}=\alpha \left(1-f_{X}(t)\right)X_{1,a}-\left(\gamma_{V,a,U}+\tau_{a}\right)H_{1,a,U} & \\ {\dot{H}}_{k,a,U}=\alpha \left(1-f_{X}(t)\right)X_{k,a}+\gamma_{V,a,U}\left(H_{k-1,a,U}-H_{k,a,U}\right)-\tau_{a}H_{k,a,U} & \text{for }k=2,...,m_{a,T}\\ {\dot{H}}_{k,a,U}=\alpha X_{k,a}+\gamma_{V,a,U}\left(H_{k-1,a,U}-H_{k,a,U}\right)-\tau_{a}H_{k,a,U} & \text{for }k=m_{a,T}+1,...,m \end{array}$

### Contact rates and basic reproduction number

#### Contact matrix

For the mixing of the age classes, we employ a who-acquires-infection-from whom matrix $M=(m_{a_{s},a_{i}})$ which gives the relative frequency of contacts of infective individuals of age *a*_*i *_with other people of age *a*_*s*_. In this paper, we assume bi-directional contacts (e.g. children have the same total number of contacts with adults as adults with children). Multiplication of this matrix with an appropriate constant scaling factor *κ *(see below) results in the matrix of crude contact rates $\beta_{a_{s},a_{i}}=\kappa \;m_{a_{s},a_{i}}$.

#### Contagiousness of the different types of disease

In the absence of interventions, we have to multiply these contact rates with the contagiousness factors *b*_*L*_, *b*_*A*_, *b*_*M *_and *b*_*V *_to obtain the effective contact rates:

$\beta_{L,a_{s},a_{i}}=b_{L}\beta_{a_{s},a_{i}}$   during the early infectious period,

$\beta_{A,a_{s},a_{i}}=b_{A}\beta_{a_{s},a_{i}}$   of asymptomatic cases,

$\beta_{M,a_{s},a_{i}}=b_{M}\beta_{a_{s},a_{i}}$   of moderately sick cases,

$\beta_{V,a_{s},a_{i}}=b_{V}\beta_{a_{s},a_{i}}$   of (untreated) very sick cases.

#### Day care centres and schools

To assess the effect of day care centre and school closing on the transmission of an infectious disease, we have to first make an assumption on what fraction *r*_*sch *_of the contacts among healthy children who are in the same age class occurs in day care centres and schools. The contact rates between very sick or hospitalized children (who do not attend day care centre or school) and other children need, therefore, be reduced to ${\beta^{\prime }}_{V,a_{s},a_{i}}(t)=(1-r_{sch})\beta_{V,a_{s},a_{i}}$ (contact rate between healthy and very sick children in the same age class, i.e. *a*_*i *_= *a*_*s*_).

As very sick children have to be taken care of by adults at home or in hospital, their contact rate to adults increases by a factor ${\beta^{\prime }}_{V,a_{s},a_{i}}(t)=\beta_{V,a_{s},a_{i}}$*F*_*HC *_(contact rate between very sick children of age *a*_*i *_and adults of age *a*_*s*_).

Contacts between very sick children and other children in a higher or lower age class remain unchanged: ${\beta^{\prime }}_{V,a_{s},a_{i}}(t)=\beta_{V,a_{s},a_{i}}$ (contact rate between healthy children of age *a*_*s *_and very sick children of a different age *a*_*i*_).

#### Closing of day care centres and schools

Closing day care centres and schools at time *t *will not necessarily prevent all the contacts that would have happened with other children. During the closing of schools and day care centres, the contact rates between susceptible children of age *a*_*s *_and infected children of age *a*_*i *_who are in their late incubation period ($\beta_{L,a_{s},a_{i}}$), who are asymptomatic ($\beta_{A,a_{s},a_{i}}$), or who are moderately sick ($\beta_{M,a_{s},a_{i}}$) are reduced by the factor *r*_*sch *_if the children are in the same age class:

$\begin{array}{l} {\beta^{\prime }}_{L,a_{s},a_{i}}(t)=\{\begin{array}{ll} \beta_{L,a_{s},a_{i}}{\left(1-r_{sch}\right)}^{1_{sch}(t)} & if\;a_{s}=a_{i}\\ \beta_{L,a_{s},a_{i}} & if\;a_{s}\neq a_{i}, \end{array}\\ {\beta^{\prime }}_{A,a_{s},a_{i}}(t)=\{\begin{array}{ll} \beta_{A,a_{s},a_{i}}{\left(1-r_{sch}\right)}^{1_{sch}(t)} & if\;a_{s}=a_{i}\\ \beta_{A,a_{s},a_{i}} & if\;a_{s}\neq a_{i}, \end{array}\\ {\beta^{\prime }}_{M,a_{s},a_{i}}(t)=\{\begin{array}{ll} \beta_{M,a_{s},a_{i}}{\left(1-r_{sch}\right)}^{1_{sch}(t)} & if\;a_{s}=a_{i}\\ \beta_{M,a_{s},a_{i}} & if\;a_{s}\neq a_{i}. \end{array} \end{array}$

where **1**_*sch *_(*t*) is a function which indicates when schools and day care centres are opened or closed:

$1_{sch}(t)=\left\{ \begin{array}{l} 1⥄⥄⥄\text{while day care centres and schools are closed}\\ 0⥄⥄⥄\text{while day care centres and schools are opened}\text{.} \end{array}\right.$

While day care centres and schools are closed, children (age *a*_*i*_) need adult supervision at home. Their contact with susceptible adults (age *a*_*s*_) increases by the "child care factor" *F*_*CC*_:

$\begin{array}{l} {\beta^{\prime }}_{L,a_{s},a_{i}}(t)=\beta_{L,a_{s},a_{i}}{\left(F_{CC}\right)}^{1_{sch}(t)},\\ {\beta^{\prime }}_{A,a_{s},a_{i}}(t)=\beta_{A,a_{s},a_{i}}{\left(F_{CC}\right)}^{1_{sch}(t)},\\ {\beta^{\prime }}_{M,a_{s},a_{i}}(t)=\beta_{M,a_{s},a_{i}}{\left(F_{CC}\right)}^{1_{sch}(t)}, \end{array}$

Child care at home also increases the exposure of healthy children (age *a*_*s*_) to contagious adults (age *a*_*i*_):

$\begin{array}{l} {\beta^{\prime }}_{L,a_{s},a_{i}}(t)=\beta_{L,a_{s},a_{i}}{\left(F_{CC}\right)}^{1_{sch}(t)},\\ {\beta^{\prime }}_{A,a_{s},a_{i}}(t)=\beta_{A,a_{s},a_{i}}{\left(F_{CC}\right)}^{1_{sch}(t)},\\ {\beta^{\prime }}_{M,a_{s},a_{i}}(t)=\beta_{M,a_{s},a_{i}}{\left(F_{CC}\right)}^{1_{sch}(t)},\\ {\beta^{\prime }}_{V,a_{s},a_{i}}(t)=\beta_{V,a_{s},a_{i}}{\left(F_{CC}\right)}^{1_{sch}(t)}. \end{array}$

#### Cancelling of mass gathering events

Cancelling mass gathering events effects only the contacts of adults who are healthy enough to attend such events. Assuming that such an intervention at time *t *reduces contacts by a fraction *r*_*mass*_, we get for all contacts between susceptible adults of age *a*_*s *_and infectious adults of age *a*_*i *_the following contact rates:

$\begin{array}{l} {\beta^{\prime }}_{L,a_{s},a_{i}}(t)=\beta_{L,a_{s},a_{i}}{\left(1-r_{mass}\right)}^{1_{mass}(t)},\\ {\beta^{\prime }}_{A,a_{s},a_{i}}(t)=\beta_{A,a_{s},a_{i}}{\left(1-r_{mass}\right)}^{1_{mass}(t)},\\ {\beta^{\prime }}_{M,a_{s},a_{i}}(t)=\beta_{M,a_{s},a_{i}}{\left(1-r_{mass}\right)}^{1_{mass}(t)}. \end{array}$

where **1**_*mass *_(*t*) is a function which indicates when mass gathering events are possible or when they are closed:

$1_{mass}(t)=\left\{ \begin{array}{l} 1⥄⥄⥄\text{while mass gathering events are forbidden}\\ 0⥄⥄⥄\text{while mass gathering events are allowed}\text{.} \end{array}\right.$

As contacts with adults who are too sick to attend such mass gathering events cannot be prevented by this measure it is

${\beta^{\prime }}_{V,a_{s},a_{i}}(t)=\beta_{V,a_{s},a_{i}}$.

#### General reduction of contacts

During some time in the epidemic, the general population may effectively reduce contacts which can be a result of wearing facial masks, increasing "social distance", adopting improved measures of "respiratory hygiene" or simply of a general change in behaviour. This will be implemented in the program by reducing the contacts of susceptible individuals at that time *t *by factor *r*_*gen *_(*t*). The adjusted contact rates are:

${\beta^{\prime \prime }}_{L,a_{s},a_{i}}(t)={\beta^{\prime }}_{L,a_{s},a_{i}}(t){\left(1-r_{gen}\right)}^{1_{gen}(t)}$   for cases in the late incubation period,

${\beta^{\prime \prime }}_{A,a_{s},a_{i}}(t)={\beta^{\prime }}_{A.a_{s},a_{i}}(t){\left(1-r_{gen}\right)}^{1_{gen}(t)}$   for asymptomatic cases,

${\beta^{\prime \prime }}_{M,a_{s},a_{i}}(t)={\beta^{\prime }}_{M.a_{s},a_{i}}(t){\left(1-r_{gen}\right)}^{1_{gen}(t)}$   for moderately sick cases,

${\beta^{\prime \prime }}_{V,a_{s},a_{i}}(t)={\beta^{\prime }}_{V,a_{s},a_{i}}(t){\left(1-r_{gen}\right)}^{1_{gen}(t)}$   for very sick cases,

where **1**_*gen *_(*t*) is a function which indicates when the population reduces their contacts:

$1_{gen}(t)=\left\{ \begin{array}{l} 1⥄⥄⥄\text{while the population reduces their contacts}\\ 0⥄⥄⥄\text{while the population behaves as usual}\text{.} \end{array}\right.$

#### Partial isolation of cases

If cases are (partly) isolated, their contact rates are reduced by factors $\left(1-r_{iso_{M}}\right)$, $\left(1-r_{iso_{V}}\right)$ and $\left(1-r_{iso_{H}}\right)$, respectively, resulting in contact rates

${\beta^{\prime \prime \prime }}_{M,a_{s},a_{i}}(t)={\beta^{\prime \prime }}_{M,a_{s},a_{i}}(t){\left(1-r_{iso_{M}}\right)}^{1_{iso}(t)}$   for moderately sick cases,

${\beta^{\prime \prime \prime }}_{V,a_{s},a_{i}}(t)={\beta^{\prime \prime }}_{V,a_{s},a_{i}}(t){\left(1-r_{iso_{V}}\right)}^{1_{iso}(t)}$   for very sick cases at home,

${\beta^{\prime \prime \prime }}_{H,a_{s},a_{i}}(t)={\beta^{\prime \prime }}_{V,a_{s},a_{i}}(t){\left(1-r_{iso_{H}}\right)}^{1_{iso}(t)}$   for hospitalized very sick cases,

where **1**_*iso *_(*t*) is a function which indicates when mass gathering events are possible or when they are closed:

$1_{iso}(t)=\left\{ \begin{array}{l} 1⥄⥄⥄\text{while isolation measures are performed}\\ 0⥄⥄⥄\text{while no isolation measures are performed}\text{.} \end{array}\right.$

The contact rates of cases in the late incubation period and that of asymptomatic cases remain unchanged:

${\beta^{\prime \prime \prime }}_{L,a_{s},a_{i}}(t)={\beta^{\prime \prime }}_{L,a_{s},a_{i}}(t)$   for infected individuals in the late incubation period,

${\beta^{\prime \prime \prime }}_{M,a_{s},a_{i}}(t)={\beta^{\prime \prime }}_{A,a_{s},a_{i}}(t)$   for asymptomatic cases.

#### Course of contagiousness

To allow for a contagiousness which changes over the course of disease, we multiply each contact rate with a weighting factor $p_{k}=x^{k-1}/\sum_{i=0}^{m-1}x^{i}$ whereby *k *is the stage of contagiousness. This leads to the following contact rates:

$\beta_{A_{k},a_{s},a_{i}}(t)={\beta^{\prime \prime \prime }}_{A,a_{s},a_{i}}(t)p_{k}$   for asymptomatic cases in stage *k*,

$\beta_{M_{k},a_{s},a_{i}}(t)={\beta^{\prime \prime \prime }}_{M,a_{s},a_{i}}(t)p_{k}$   for moderately sick cases in stage *k*,

$\beta_{V_{k},a_{s},a_{i}}(t)={\beta^{\prime \prime \prime }}_{V,a_{s},a_{i}}(t)p_{k}$   for very sick cases in stage *k*,

$\beta_{H_{k},a_{s},a_{i}}(t)={\beta^{\prime \prime \prime }}_{H,a_{s},a_{i}}(t)p_{k}$   for hospitalized cases in stage *k*.

For *x *= 1, contagiousness is equally high in all stages; for *x *= 0, only the first stage is contagious; for 0 <*x *< 1, the contagiousness decreases in a geometric procession. We make the simplifying assumption that contagiousness does not change during the late incubation period

$\beta_{L_{k},a_{s},a_{i}}(t)={\beta^{\prime \prime \prime }}_{L,a_{s},a_{i}}(t)$ for cases in stage *k *= *n *- *l*,..,*n *of the incubation period.

### Next generation matrix and basic reproduction number

At time *t *= 0 and in the absence of interventions, the next generation matrix has the following elements

$n_{a_{s},a_{i}}=\left(\frac{1}{n}\sum_{k=n-l+1}^{n}\beta_{L_{k},a_{s},a_{i}}(0)D_{E}+\frac{1}{m}\sum_{r}\sum_{k=1}^{m}\left(\begin{array}{l} c_{a_{i},r}(A)\beta_{A_{k},a_{s},a_{i}}(0)D_{A,a_{i}}\\ +c_{a_{i},r}(M)\beta_{M_{k},a_{s},a_{i}}(0)D_{M,a_{i}}\\ +\left(c_{a_{i},r}(V)+c_{a_{i},r}(X)(1-d_{a_{i}})\right)\beta_{V_{k},a_{s},a_{i}}(0)D_{V,a_{i}} \end{array}\right)\right)$

where $d_{a_{i}}$ is the fraction of untreated extremely severe cases who die from the disease (see below for details). The dominant eigenvalue of this matrix is called the basic reproduction number *R*_0_. If *κ *(which determines the value of the contact rates $\beta_{{•}_{k},a_{s},a_{i}}$) is given, the eigenvectors of this matrix can numerically be calculated. The user-specified value of *R*_0 _is now used to determine numerically the scaling factor *κ*. Let $\vec{e}=(e_{a_{i}})$ be the eigenvector which has the largest eigenvalue *R*_0_.

#### Force of infection

To calculate the force of infection $\lambda_{a_{s}}$ to which susceptible individuals of age *a*_*s *_are exposed at time *t*, we have to first calculate the product of the number of contagious individuals with the corresponding contact rates and then to sum up these products over all ages *a*_*i*_, all risk categories *r*, all courses of the disease and all stages. Assuming that the contagiousness of cases who have received antiviral treatment is reduced by the factor (1 - *f*_*C*_), the force of infection is given by

$\lambda_{a_{s}}(t)=\sum_{a_{i}}\left(\sum_{r}\sum_{k=n-l+1}^{n}\beta_{L_{k},a_{s},a_{i}}(t)E_{k,a_{i},r}+\sum_{k=1}^{m}\left(\begin{array}{l} \beta_{A_{k},a_{s},a_{i}}(t)A_{k,a_{i}}+\beta_{M_{k},a_{s},a_{i}}(t)⥄M_{k,a_{i}}\\ +\beta_{V_{k},a_{s},a_{i}}(t)\left(V_{k,a_{i}}+W_{k,a_{i},U}+(1-f_{I})W_{k,a_{i},T}+X_{k,a_{i}}\right)\\ +\beta_{H_{k},a_{s},a_{i}}(t)\left(H_{k,a_{i},U}+(1-f_{I})H_{k,a_{i},T}\right) \end{array}\right)\right)$

#### Differential equations for various model output

Cumulative number of deaths

$\dot{D}=\sum_{a}\sum_{k=1}^{m}\left(\tau_{a,U}\left(X_{a}+H_{k,a,U}\right)+\tau_{a}H_{k,a,T}\right)$

Convalescent (but non-contagious) cases

$\begin{array}{cc} \begin{array}{l} {\dot{C}}_{1,a}=\gamma_{V,a,U}\left(V_{m,a}+W_{m,a,U}+X_{m,a}+H_{m,a,U}\right)+\gamma_{V,a,T}\left(V_{m,a,T}+H_{m,a,T}\right)-\rho C_{1,a}\\ {\dot{C}}_{k,a}=\rho (C_{k-1,a}+C_{k,a}) \end{array} & \text{for }k=2,...,j \end{array}$

Immune and fully recovered individuals

$\dot{I}=\sum_{a}\left(\rho C_{j,a}+\gamma_{A}A_{m,a}+\gamma_{M}M_{m,a}\right)$

Number of people who are unable to work because of influenza

$\dot{U}=\sum_{a_{W}}\left(\sum_{r}\delta E_{n,a_{W},r}(c_{a_{W},r}(V)+c_{a_{W},r}(X))-\tau_{a_{W}}\sum_{k=1}^{m}\left(X_{k,a_{W}}+H_{k,a_{W},U}+H_{k,a_{W},T}\right)-\rho C_{j,a_{W}}\right)$

where *a*_*W *_denote all age classes of working adults (to avoid infinite contributions to the work loss, the decision was made that cases who die from influenza do not contribute any further to the total work loss).

Cumulative doses of antiviral treatment

$\dot{T}=\alpha \sum_{k=1}^{m_{a,T}}\sum_{a}\left(f_{V}(t)V_{k,a}+f_{X}(t)X_{k,a}\right)$

#### Initial values

Using the user-specified numbers of people *N*_*a *_in the age classes and the fractions *F*_*a *_of people under high risk within each age class (Table 2), we obtain the initial population sizes according to age and risk class: $N_{a,r_{1}}$ (0) = *N*_*a *_(1 - *F*_*a*_) and $N_{a,r_{2}}$ (0) = *N*_*a*_*F*_*a*_. The total population is, therefore, given by $N(0)=\sum_{a}\sum_{r}N_{a,r}(0)$.

At time *t *= 0, one infection is introduced into an otherwise fully susceptible population. To avoid biasing the simulation one way or the other, the initial infection is distributed over all classes, weighted by the probability that an individual in one class acquires the infection (i.e. by the component of the eigenvector $\vec{e}=(e_{a})$ of the next generation matrix):

$S_{a,r}(0)=N_{a,r}(0)-\{\begin{array}{ll} (1-F_{r}){\text{e}}_{a}/\sum_{a_{i}}{\text{e}}_{a_{i}} & \text{if }r=r_{1}\text{ (low risk group)}\\ F_{r}{\text{e}}_{a}/\sum_{a_{i}}{\text{e}}_{a_{i}} & \text{if }r=r_{2}\text{ (high risk group)} \end{array}$

$E_{k,a,r}(0)=\{\begin{array}{ll} (1-F_{r})F_{r}{\text{e}}_{a}/\sum_{a_{i}}{\text{e}}_{a_{i}} & \text{if }r=r_{1}\text{ (low risk group) and }k=1\\ F_{r}{\text{e}}_{a}/\sum_{a_{i}}{\text{e}}_{a_{i}} & \text{if }r=r_{2}\text{ (high risk group) and }k=1\\ 0 & \text{if }k>1 \end{array}$

$\forall_{k=1}^{m}$*A*_*k*, *a *_(0)* = M*_*k*, *a *_(0) = *V*_*k*, *a *_(0) = *W*_*k*, *a*, *U *_(0) = *W*_*k*, *a*, *T *_(0) = *X*_*k*, *a *_(0) = *H*_*k*, *a*, *U *_(0) = *H*_*k*, *a*, *T *_(0) = 0

$\forall_{k=1}^{j}$*C*_*k*, *a *_(0) = 0, *D *(0) = *I *(0) = *U *(0) = *T *(0) = 0.

Using these initial values, the set of differential equations is solved numerically with a Runge-Kutta method with step-size control.

## Abbreviations

### Model variables

#### Transmission variables

*S*_*a*, *r *_number of susceptible individuals

*E*_*k*, *a*, *r *_number of incubating individuals (stage *k*); the last two stages are contagious

*A*_*k*, *a *_number of asymptomatic individuals (stage *k*)

*M*_*k*, *a *_number of moderately sick individuals (stage *k*)

*V*_*k*, *a *_number of very sick individuals who have not yet seen a doctor (stage *k*)

*W*_*k*, *a*, *T *_number of treated very sick individuals (withdrawn to home; stage *k*)

*W*_*k*, *a*, *U *_number of untreated very sick individuals (withdrawn to home; stage *k*)

*X*_*k*, *a *_number of extremely sick individuals who have not seen a doctor (stage *k*)

*H*_*k*, *a*, *T *_number of hospitalized and treated individuals (stage *k*)

*H*_*k*, *a*, *U *_number of hospitalized but untreated individuals (stage *k*)

#### Output variables

*C*_*k*, *a *_number of convalescent (non-contagious) cases (stage *k*)

*I *number of fully recovered and immune cases

*D *number of people who die of influenza

*U *number of people who are unable to work because of influenza

*T *cumulative number of antiviral treatment doses used

#### Parameters concerning the demography

*N*_*a *_total population size by age class *a*, whereby *a *= *a*_1 _denotes children, *a *= *a*_2 _denotes adults of working age and *a *= *a*_2 _denotes elderly, respectively.

*F*_*a *_fraction of the population in age class *a *which is under high risk from this, *N*_*a*, *r *_is calculated such that *N*_*a*, *r *_= *F*_*a*_*r*_*a*_

$K_{a_{s},a_{i}}$ the contact matrix gives the weekly number of contacts between an individual of age class *a*_*i *_with individuals of age class *a*_*s*_. From this, the contact rates $\beta_{L_{k},a_{s},a_{i}}(t)$, $\beta_{A_{k},a_{s},a_{i}}(t)$, $\beta_{M_{k},a_{s},a_{i}}(t)$ and $\beta_{V_{k},a_{s},a_{i}}(t)$ are calculated as explained above

### Parameters concerning the natural history of the disease

#### Number of stages

*n *number of stages used to model the latent period

*l *number of stages used to model the early infectious period

*m *number of stages used to model the (symptomatic) infectious period

*j *number of stages used to model convalescence

#### Sojourn times

*D*_*E *_average duration of the incubation period;

*δ *is calculated such that *δ *= *n*/*D*_*E*_

the last *l *stages are used as early infectious period

(average duration: *D*_*L *_= *D*_*E*_*l*/*n*)

*D*_*D *_average time after onset when a severe case seeks medical help;

*α *is calculated such that *α *= 1/*D*_*D*_

*D*_*A*, *a *_average infectious duration for asymptomatic cases

*γ*_*A*, *a *_is calculated such that *γ*_*A*, *a *_= *m*/*D*_*A*, *a*_

*D*_*M*, *a *_average infectious duration of moderately sick cases

*γ*_*M*, *a *_is calculated such that *γ*_*M*, *a *_= *m*/*D*_*M*, *a*_

D_*V*, *a *_average duration of infectivity of untreated very or extremely sick cases;

*γ*_*V*, *a*, *U *_is calculated such that *γ*_*V*, *a*, *U *_= *m*/*D*_*V*, *a*_

*D*_*C *_average duration of convalescence;

*ρ *is calculated such that *ρ *= *j*/*D*_*C*_

#### Course of disease

*c*_*a*, *r *_(*A*) fraction of asymptomatic infections (given age *a *and risk *r*)

*s*_*a*, *r *_fraction of severe cases among symptomatic ones

*h*_*a*, *r *_fraction of severe cases who need hospitalization (unless treated) the fraction of infected cases who

- develops moderate disease is *c*_*a*, *r *_(*M*) = (1 - *s*_*a*, *r*_)(1 - *c*_*a*, *r *_(*M*))

- becomes bed-ridden at home is *c*_*a*, *r *_(*V*) = *s*_*a*, *r *_(1 - *h*_*a*, *r*_)(1 - *c*_*a*, *r *_(*M*))

- become extremely severe cases is *c*_*a*, *r *_(*X*) = *s*_*a*, *r*_*h*_*a*, *r *_(1 - *c*_*a*, *r *_(*M*))

*d*_*a *_fraction of untreated extremely severe cases who die;

from this, *τ*_*a *_is chosen such that $d_{a}=\frac{\tau_{a}}{\tau_{a}+\gamma_{S,a,U}}\sum_{k=0}^{m-1}{\left(\frac{\tau_{a}}{\tau_{a}+\gamma_{S,a,U}}\right)}^{k}$

### Parameters concerning the contagiousness of the infection

*b*_*L *_relative contagiousness of cases in the late incubation period

*b*_*A *_relative contagiousness of asymptomatic cases

*b*_*M *_relative contagiousness of moderately sick cases

*b*_*V *_relative contagiousness of severely sick cases

*x*_50 _parameter regulating the course of contagiousness

*x*_50 _= 1 only the first stage after onset of disease is contagious

0.5 <*x*_50 _< 1 contagiousness decreases after onset of disease

*x*_50 _= 0.5 equal contagiousness during the whole course of disease

0 <*x*_50 _< 0.5 contagiousness increases after onset of disease

from this, *x *is calculated such that $x_{50}=\sum_{i=0}^{m/2}x^{i-1}/\sum_{i=0}^{m}x^{i-1}$ if *m *is an even number or $x_{50}=\left(\sum_{i=0}^{(m-1)/2}x^{i-1}+\frac{x^{\left(m-1\right)/2+1}}{2}\right)/\sum_{i=0}^{m}x^{i-1}$ if *m *is an odd number, respectively

*R*_0 _basic reproduction number; the contact rates $\beta_{L_{k},a_{s},a_{i}}(t)$, $\beta_{A_{k},a_{s},a_{i}}(t)$, $\beta_{M_{k},a_{s},a_{i}}(t)$ and $\beta_{V_{k},a_{s},a_{i}}(t)$ are calculated from *R*_0 _and from the contagiousness factors as explained above

*λ*_*a *_(*t*) force of infection for susceptible individuals of age *a *at time *t *(see calculation above)

### Parameters concerning contact reduction

$r_{iso_{M}}$ fraction of contacts of moderately sick patients that are prevented by partial isolation

$r_{iso_{V}}$ fraction of contacts of very sick patients that are prevented by partial isolation

$r_{iso_{H}}$ fraction of contacts of hospitalized patients that are prevented by partial isolation

*r*_*gen *_general fraction of contacts that are prevented at time *t*

*r*_*mass *_fraction of contacts among (healthy) adults that are prevented by cancelling events of mass gatherings at time *t*

*r*_*sch *_fraction of contacts among (healthy) children of the same age class that occurs in day care centres or schools

*F*_*HC *_factor by which the contacts between adults and severely sick children increase because of child health care

*F*_*CC *_factor by which the contacts between adults and children increase when children are taken care off at home because schools are closed

### Parameters concerning antiviral treatment

*T*_max _available number of antiviral treatment doses

*D*_*T *_time after onset until when antiviral treatment can still be given; the latest infectious stage *m*_*a*, *T *_during which treatment can be given, is chosen such that *m*_*a*, *T*_/*γ*_*V*, *a*, *U *_≤ *D*_*T *_≤ (*m*_*a*, *T *_+ 1)/*γ*_*V*, *a*, *U*_

*f*_*V *_fraction of severe cases eligible to receive antiviral treatment; treatment will be given only in the user-specified time window and only as long as supplies last:

$f_{V}(t)=\{\begin{array}{ll} f_{V} & \text{if }T(t)<T_{\max}\text{ and }t\text{ in treatment window}\\ 0 & \text{otherwise} \end{array}$

*f*_*X *_fraction of extremely severe cases eligible to receive antiviral treatment; treatment will be given only in the user-specified time window and only as long as supplies last:

$f_{X}(t)=\{\begin{array}{ll} f_{X} & \text{if }T(t)<T_{\max}\text{ and }t\text{ in treatment window}\\ 0 & \text{otherwise} \end{array}$

*f*_*D *_fraction by which the duration of infectiousness is reduced by antivirals; *γ*_*V*, *a*, *T *_is calculated from this such that *γ*_*V*, *a*, *T *_= *m*/((1 - *f*_*D*_)*D*_*V*, *a*_)

*f*_*I *_fraction by which the infectiousness of treated cases is reduced by antivirals

*f*_*H *_fraction of hospitalizations prevented by antiviral treatment

## Pre-publication history

The pre-publication history for this paper can be accessed here:

http://www.biomedcentral.com/1471-2334/7/17/prepub
